# NMR Studies on Protein–Ligand Interactions

**DOI:** 10.3390/ijms27177561

**Published:** 2026-08-24

**Authors:** Haiqin Yao, Ning Xu

**Affiliations:** 1 School of Life Sciences, Tsinghua University, Beijing 100084, China; 2 Technology Center for Protein Sciences, Tsinghua University, Beijing 100084, China

**Keywords:** protein–ligand interactions, NMR screening, interface mapping, solution structures, conformational dynamics

## Abstract

Protein–ligand interactions are fundamental to physiological processes and drug discovery. Based on the types of information on protein–ligand interactions provided by nuclear magnetic resonance (NMR) experiments, these NMR experiments can be categorized into three distinct classes: (i) molecular-level qualitative binding detection, (ii) residue-level mapping of binding interfaces, and (iii) atomic-level structure determination and conformational dynamics of protein–ligand complexes. This hierarchy enables a workflow that accelerates the progression from initial binding identification to structural and dynamic characterization. In this review, we discuss how these experiments characterize molecular recognition. Notably, the term “ligand” in this article refers exclusively to small molecules.

## 1. Introduction

Advances in metabolomics continue to identify new metabolites that are involved in physiological regulation [1]. Concurrently, artificial intelligence (AI)-driven virtual screening enables the exploration of chemical compound libraries that far exceed the size of traditional high-throughput screening collections [2]. These advances have provided new insights into protein–ligand interactions, bringing both opportunities and challenges: numerous small molecules ranging from metabolites to drug candidates require experimental characterization to validate their binding to proteins of interest. Determining whether and how these small molecules interact with target proteins is therefore critical. Many techniques have been developed to address this need, including isothermal titration calorimetry (ITC), surface plasmon resonance (SPR), and others [3,4]. Over the past few decades, driven by cryogenic NMR probe technology, NMR has emerged as a powerful tool for investigating protein–ligand interactions. It is capable of detecting and characterizing binding events across a wide affinity range, extending from millimolar to nanomolar dissociation constants (*K*_d_) [5,6,7].

According to the types of information on protein–ligand interactions derived from NMR experiments, NMR provides a continuous view of molecular recognition that bridges qualitative screening and atomic-resolution structural and dynamic descriptions of protein–ligand interactions. This multi-scale capability is manifested across three levels:

First, at the molecular level, NMR serves as a rapid and sensitive filter to address the most fundamental question: do two specific molecules (protein and ligand) interact? This capability is primarily realized through three types of ligand-observed NMR experiments, including saturation transfer difference (STD), water–ligand observed via gradient spectroscopy (WaterLOGSY), and relaxation-based approaches such as spin–lattice relaxation in the rotating frame (T_1ρ_) and Carr–Purcell–Meiboom–Gill (CPMG) experiments [8,9,10]. Ideally suited for screening compound mixtures with minimal protein consumption, these techniques collectively offer a rapid and reliable strategy to discriminate binders from non-binders.

Second, at the residue level, NMR not only addresses the fundamental question of whether a specific protein–ligand interaction exists but also identifies which residues are involved in the binding interfaces. NMR maps binding interfaces by monitoring changes in the protein itself. A commonly used experiment is the ^1^H-^15^N heteronuclear single quantum coherence (HSQC), which detects binding-induced chemical shift perturbation (CSP) to characterize interaction sites [11]. For challenging systems such as large macromolecular complexes or membrane proteins that exceed the limits of conventional ^1^H-^15^N HSQC, site-specific labeling strategies are essential [12,13]. ^13^C-methyl labeling serves as a protein-observed approach for intractable protein–ligand interactions [14]. Due to its exceptional sensitivity and lack of background signals, ^19^F NMR enables application in both protein-observed and ligand-observed modes [15]. Together, these two site-specific labeling strategies play important roles in studies on protein–ligand interactions, particularly in research related to G protein-coupled receptors (GPCRs) [16,17].

Finally, at the atomic level, NMR not only confirms the existence of specific interactions and maps their interfaces but also elucidates the precise molecular recognition mechanisms. To achieve this, NMR facilitates the determination of three dimensional solution structures of protein–ligand complexes [18]. This step provides a detailed structural model of the recognition interface, while NMR simultaneously captures conformational dynamics, collectively converting qualitative binding data into mechanistic insights.

Herein, by integrating these hierarchical and progressive experiments, we elucidate how these methodologies work to confirm and characterize protein–ligand interactions. These approaches constitute an indispensable toolkit for fully mapping the landscape of protein–ligand interactions. We note that this review focuses on the interactions between proteins and small-molecule ligands; interactions of proteins with other biomolecules, such as other proteins or nucleic acids, are equally important but involve distinct experimental considerations and are beyond the scope of this article.

## 2. Ligand-Observed NMR: Tools for Rapid Binding Detection

### 2.1. Saturation Transfer Difference (STD)

As a ligand-observed NMR experiment, STD is used to detect protein–ligand interactions with dissociation constants (K_d_) ranging from millimolar to micromolar [8,19]. In the STD experiment, protein protons are selectively saturated by irradiating a spectral region that excludes ligand signals. This saturation spreads throughout the entire protein via dipole–dipole interactions, a process known as spin diffusion [20]. If the ligand binds to the protein, the saturation is transferred from the protein to the ligand protons. The bound ligand then dissociates into solution, carrying the saturated protons with it. The cycle of binding and dissociation between protein and ligand repeats during the STD experiment, leading to an accumulation of saturation on the ligand. Thus, a critical requirement for the STD experiment is that the ^1^H NMR signals of the ligand and the protein do not overlap in the spectral region where selective saturation is applied [19].

**Figure 1:**
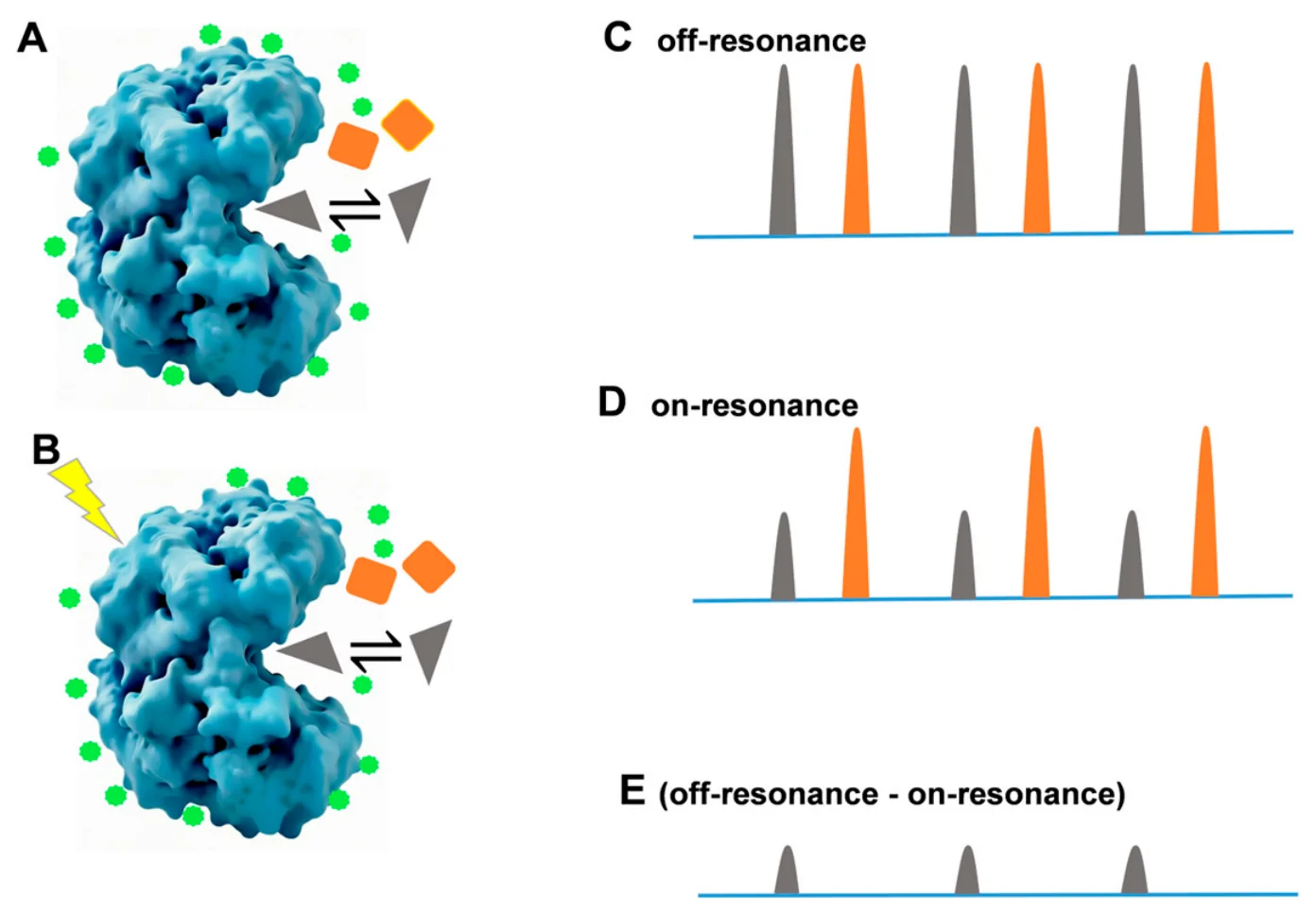
STD analysis confirms the grey ligand interacts with the protein. (**A**) Schematic of the protein–ligand complex without selective saturation (blue: protein, grey/orange: ligands, green: water). (**B**) Schematic of the protein–ligand complex under selective saturation (blue: protein; grey/orange: ligands; green: water, yellow: selective saturation irradiation at proper frequency). (**C**) Off-resonance spectrum under condition A. (**D**) On-resonance spectrum under condition B. (**E**) The STD difference spectrum (off-resonance—on-resonance) shows signals only for the grey ligand, confirming its direct interaction with the protein, while the orange ligand shows no binding.

The STD experiment consists of two interleaved scans. One is the on-resonance scan. The protein protons are irradiated, resulting in signal attenuation for the bound ligand in the STD experiment. The other is the off-resonance scan. The saturation pulse is applied far from any resonances, providing a reference spectrum with full signal intensity for all compounds. The difference spectrum (off-resonance spectrum minus on-resonance spectrum) reveals signals only from molecules that bind to the target protein, whose protons are in close contact with the protein. These bound compounds receive more saturation and thus show stronger signals in the STD difference spectrum (Figure 1).

The STD experiment has been demonstrated to be highly sensitive, requiring as little as 1 nmol of protein and remaining effective for targets with molecular weights greater than 10 kDa [20]. In practice, protein concentrations are generally in the range from 20 to 50 μM, and the molar ratio of the ligand to the target protein is typically maintained between 10:1 and 100:1 [8,21]. Depending on the specific system and observed signal intensity, both the protein concentration and the ligand-to-protein ratio can be adjusted. Regarding sample conditions, either 100% D_2_O or 90% H_2_O/10% D_2_O buffers are acceptable. Using a 100% D_2_O sample requires lyophilization of the protein to exchange the solvent, which adds an extra preparation step compared to using a 90% H_2_O/10% D_2_O sample. Conversely, samples in 90% H_2_O/10% D_2_O require effective water suppression techniques during NMR data acquisition to eliminate the strong solvent signal.

In the STD pulse sequence, two critical parameters—the saturation time and the saturation power—must be carefully considered. On Bruker NMR spectrometers, saturation times are typically set between 2 and 5 s, while saturation power is typically set in the range from 40 to 60 dB. Theoretically, increasing the saturation time and applying higher saturation power should maximize the transfer of magnetization from the protein to the ligand, thereby enhancing the STD signals. However, careful optimization of saturation time and power is crucial. As demonstrated by Meyer and Mayer, utilizing low irradiation power and specific saturation times ensures selective protein saturation to avoid direct ligand excitation [20]. Furthermore, Xia et al. highlighted that power spillover from RF irradiation is a significant source of artifacts in STD experiments, particularly for smaller proteins [22]. Therefore, establishing optimal experimental conditions requires a balance between maximizing sensitivity and minimizing artifacts. In practice, the optimal saturation time and saturation power can be determined using control samples containing the ligand in the absence of proteins. In the STD experiment, intramolecular spin diffusion within the bound ligand can propagate magnetization from the binding interface to distant protons. Consequently, protons in non-binding regions of the ligand may also become saturated, which can make it difficult to precisely identify the binding site on the ligand, especially at long saturation times.

### 2.2. Water-Ligand Observed via Gradient Spectroscopy (WaterLOGSY)

The WaterLOGSY experiment is particularly well suited for detecting moderately to weakly binding ligands, typically with dissociation constants in the micromolar (μM) to low millimolar (mM) range [9]. WaterLOGSY is built upon the principle of the nuclear Overhauser effect (NOE). The sign of NOE signals is related to the molecular weight and tumbling rate of molecules. Generally, small molecules tumble rapidly in solution with short correlation times, whereas large biomolecules tumble slowly with long correlation times. Consequently, these two regimes exhibit opposite NOE behaviors. This fundamental dependence of NOE sign on molecular size forms the basis for applying WaterLOGSY to protein–ligand interactions [23].

**Figure 2:**
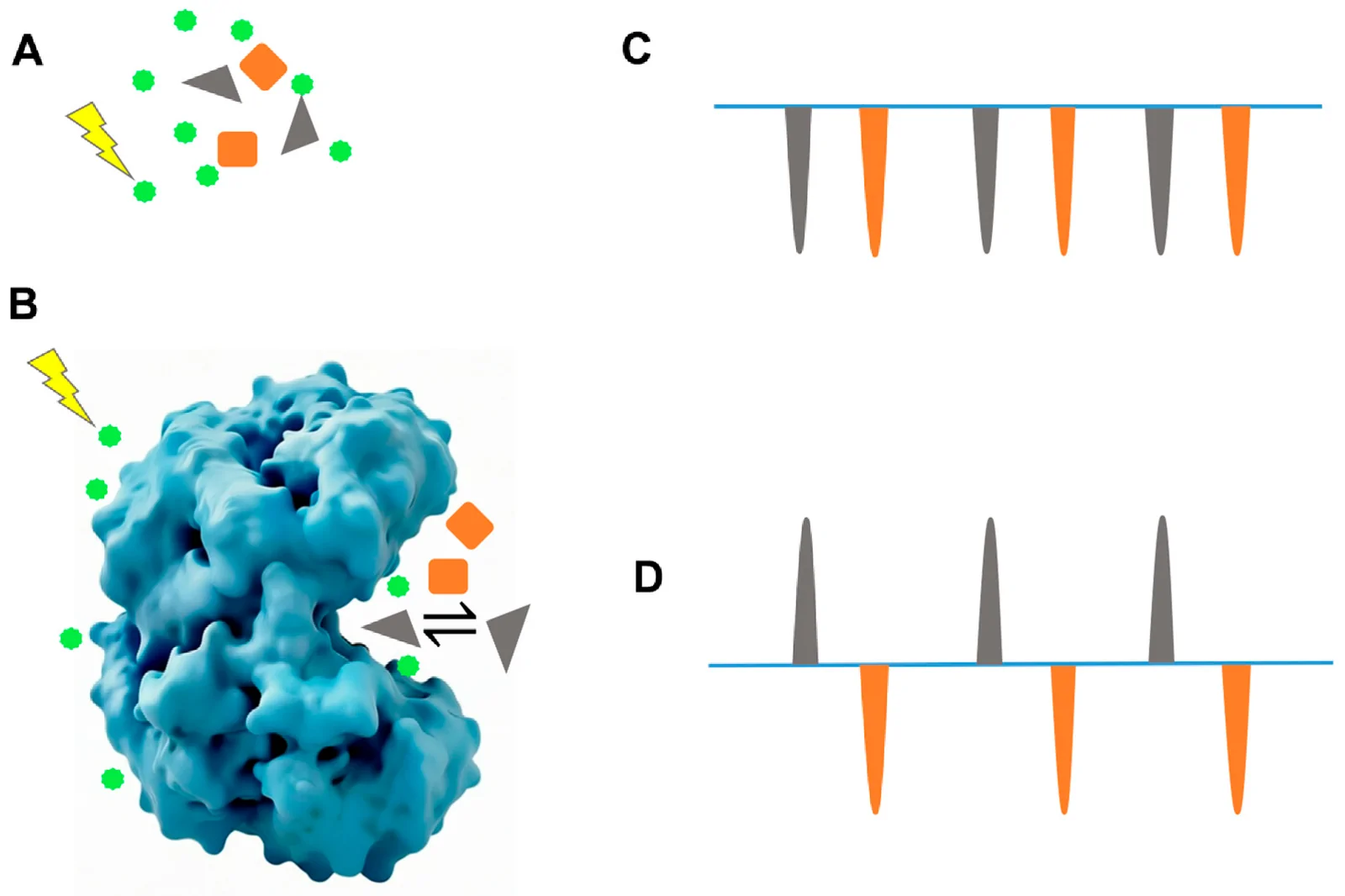
WaterLOGSY analysis confirms the grey ligand interacts with the protein. (**A**) Schematic of free ligands in solution (grey/orange: ligands; green: water, yellow: excitation of the bulk water resonance). (**B**) Schematic of ligands in the presence of the protein (blue: protein; grey/orange: ligands; green: water, yellow: excitation of the bulk water resonance). (**C**) WaterLOGSY spectrum under condition A (free ligands), showing negative signals. (**D**) WaterLOGSY spectrum under condition B (with protein); the grey ligand displays positive signals, confirming its binding, while the orange ligand remains unbound (negative peaks).

In the WaterLOGSY experiment, the magnetization of bulk water protons in the sample is selectively excited. This magnetization is subsequently transferred to small molecule ligand via two distinct pathways that show signals with opposite signs in the NMR spectrum [24]. If the ligand does not interact with the target protein in solution, it can interact directly with water molecules, and the excited magnetization is transferred directly from water to the free ligand via the NOE. Due to the fast tumbling of the free small molecule in solution, this direct NOE transfer is typically displayed as a negative signal in WaterLOGSY spectra. When a ligand binds to a protein, the excited magnetization is first transferred from water to the protein. Subsequently, it is transferred from the protein to the ligand occupying the binding site. In addition, water molecules that are bound to the protein surface (ordered water) can also participate in magnetization transfer. These bound water molecules tumble at a rate similar to the protein and can relay magnetization from the protein to the ligand or directly to the ligand via the NOE and may also exchange magnetization through chemical exchange with exchangeable protons on the protein or ligand [9]. Upon binding to the protein, the ligand’s tumbling rate becomes comparable to that of the slowly tumbling macromolecule. Under these slow tumbling conditions, the resulting NOE signal is conventionally displayed as a positive signal (Figure 2).

In practice, two sets of measurements are typically performed: a reference experiment containing only the ligand (without protein) and a screening experiment containing both the protein and the ligand. If a ligand’s signal undergoes a clear inversion from negative to positive upon the addition of the protein, it indicates that the ligand interacts with the protein. If the signal remains negative, it suggests that the ligand does not bind. It should be noted that in WaterLOGSY experiments, the observed signal of a bound ligand represents an average of the opposing NOE arising from the free and bound states [25]. This averaging means that when the free and bound contributions are similar in magnitude, the net signal can become very weak. In practice, if a ligand shows little or no signal in the screening experiment (compared to the reference), it may be due to this averaging effect rather than to poor solubility or weak binding. To test this, additional experiments with varying ligand-to-protein ratios are recommended. Changing the ligand concentration perturbs the equilibrium, altering the weighted average and allowing for the expected positive (binding) or negative (non-binding) signals to appear.

To facilitate selective excitation of the water protons, samples are generally prepared in solvent mixtures containing 90% H_2_O/10% D_2_O (or with an even lower D_2_O content). The protein concentration employed in WaterLOGSY measurements is generally in the low micromolar range (10 to 100 μM) and the ligand is typically in 25- to 100-fold excess relative to the protein concentration [9]. The mixing time for NOE buildup is usually set to 0.8 s.

### 2.3. Relaxation-Based Methods

In contrast to magnetization transfer techniques such as WaterLOGSY and STD, relaxation-based NMR methods including spin–lattice relaxation time in the rotating frame (T_1ρ_) and Carr–Purcell–Meiboom–Gill (CPMG) experiments, rely on changes in the intrinsic relaxation properties of the ligand upon binding [26]. Despite their different mechanisms, these strategies share a common physical foundation: the profound influence of molecular tumbling on NMR relaxation parameters.

**Figure 3:**
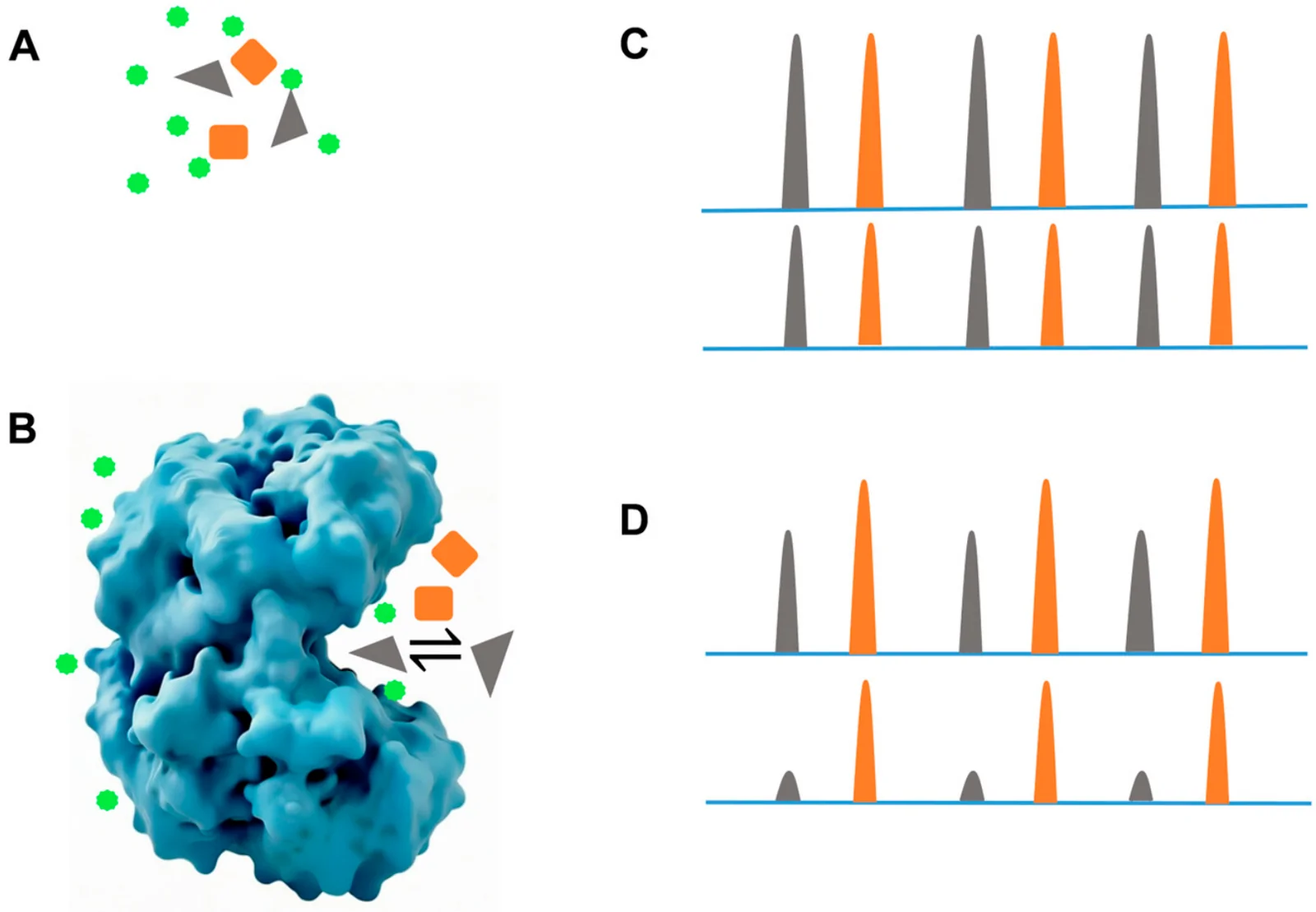
T_1ρ_ relaxation experiments confirm the grey ligand interacts with the protein. (**A**) Schematic of free ligands in solution (grey/orange: ligands; green: water). (**B**) Schematic of ligands in the presence of the protein (blue: protein; grey/orange: ligands; green: water). (**C**) T_1ρ_ spectra for free ligands (Condition A) at spin-lock times of 10 ms (top) and 200 ms (below), signals show moderate attenuation, typical of small molecules in free solution. (**D**) T_1ρ_ spectra in the presence of protein (Condition B) at spin-lock times of 10 ms (top) and 200 ms (below), signals exhibit significant reduction at 200 ms, demonstrating that the grey ligand interacts with the protein, while the orange ligand remains unbound.

The T_1ρ_ experiment exploits the dependence of spin–lattice relaxation in the rotating frame on molecular tumbling. In solution, small organic molecules tumble rapidly, characterized by a short rotational correlation time. Conversely, when a small molecule ligand binds to a large protein, the effective molecular weight of the complex increases dramatically. This slows the overall tumbling rate of the ligand nuclei, leading to a significantly longer rotational correlation time. In this slow tumbling regime, the efficiency of T_1ρ_ relaxation is greatly enhanced, causing the ligand’s NMR signals to decay rapidly, and resulting in significant line broadening. This binding-induced acceleration of relaxation forms the fundamental basis for T_1ρ_-based binding assays (Figure 3) [27].

The CPMG experiment measures the transverse relaxation rate (R_2_), a parameter highly sensitive to molecular tumbling. In the free state, rapidly tumbling small molecules experience inefficient transverse relaxation, characterized by long transverse relaxation times (T_2_). Conversely, upon binding to a macromolecular target, the ligand’s effective rotational correlation time increases significantly. This retardation of molecular motion markedly enhances transverse relaxation efficiency, thereby shortening the T_2_. The CPMG pulse sequence employs a train of refocusing pulses to attenuate signals based on their T_2_ characteristics, effectively acting as a filter that suppresses signals from slowly tumbling complexes with short T_2_ values. Consequently, ligands that interact with the protein exhibit marked signal attenuation in CPMG spectra compared to their free state, providing a robust method for identifying binders [26,28].

Both T_1ρ_ and CPMG experiments rely on the modulation of a ligand’s relaxation rates by molecular dynamics upon binding to a target macromolecule. While T_1ρ_ utilizes a continuous spin-lock field in the rotating frame to probe these dynamics, CPMG achieves a similar filtering effect in the laboratory frame through discrete refocusing pulses. Although distinct in their implementation and sensitivity profiles regarding chemical exchange, both methods serve as powerful tools for detecting ligand–protein interactions.

In practice, NMR spectra of the ligand are first recorded in the absence of the target protein at two representative spin-lock durations, for example, 10 ms and 200 ms. Then, spectra of the same ligand in the presence of the target protein under identical spin-lock conditions are collected. In both cases, signal attenuation is observed at longer spin-lock durations due to intrinsic relaxation. However, if the ligand interacts with the protein, its T_1ρ_ is significantly shortened, resulting in a greater reduction in NMR signal intensity compared to the control ligand without interaction. By comparing the signal intensity decay under these two conditions, whether a ligand binds to the target protein can be readily distinguished. An analogous workflow applies to CPMG experiments. Spectra for the ligand in the absence and presence of protein are acquired with a T_2_ filter of approximately 400 ms. The ligand binding-induced signal attenuation can then be identified.

Sample conditions for T_1ρ_ and CPMG experiments are similar to those for STD and WaterLOGSY. T_1ρ_ and CPMG experiments can adopt either 90% H_2_O/10% D_2_O or 100% D_2_O buffers. Compared with STD and WaterLOGSY, a critical distinction arises when detecting tightly binding ligands, such as those with nanomolar dissociation constants. Under conditions with a large molar excess of ligand, the limited protein binding sites become rapidly saturated. The majority of the ligand remains in the free state, causing the observed NMR signal to be dominated by unbound ligands. This phenomenon can lead to false negative results. To overcome this limitation, the ligand-to-protein ratio must be reduced, for example, to approximately 1:1. Under this condition, the observed tight binding ligand signals adopt the characteristics of the slow-tumbling protein–ligand complex and become significantly broader and attenuated. In T_1ρ_ and CPMG experiments, this distinct transition from sharp to broad signals serves as direct and robust evidence of high affinity binding.

STD, WaterLOGSY, and relaxation-based methods (T_1ρ_ or CPMG) are not subject to protein molecular weight restrictions, and their performance improves with increasing molecular weight. In practice, these methods are routinely used in combination to provide robust validation of protein–ligand interactions, significantly reducing the risk of both false positive and false negative conclusions. This combined strategy is therefore highly effective for drug screening, typically achieving a throughput of 5–10 compounds per protein sample.

## 3. Protein-Observed NMR: Strategies for Residue-Level Interface Mapping

### 3.1. ^1^H-^15^N Heteronuclear Single Quantum Coherence (HSQC)

As a protein-observed experiment, the ^1^H-^15^N HSQC experiment fundamentally utilizes heteronuclear scalar couplings to facilitate magnetization transfer, enabling the detection of low-sensitivity nuclei such as ^15^N via the high-sensitivity proton (^1^H). In the ^1^H-^15^N HSQC spectrum, one dimension corresponds to the ^1^H chemical shift, while the other dimension represents the ^15^N chemical shift. The basic pulse sequence comprises three critical components: an INEPT (Insensitive Nuclei Enhanced by Polarization Transfer) module that transfers magnetization from ^1^H to ^15^N; an evolution period during which the ^15^N chemical shift evolves; and a reverse INEPT module that transfers magnetization back from ^15^N to ^1^H for detection [29].

For well-folded proteins, the ^1^H-^15^N HSQC spectrum exhibits a highly dispersed distribution of peaks, reflecting the diverse local environments experienced by amino acid residues. In the ^1^H-^15^N HSQC spectrum, each cross peak corresponds to an amide group on the protein backbone with the exception of proline, alongside contributions from the side-chain amide groups of asparagine and glutamine, and the indole NH of tryptophan. Every amino acid in the protein sequence serves as a distinct probe capable of reporting changes in its local environment. Upon ligand binding, alterations in chemical shifts or signal intensities occur for residues near the binding interface, making this method applicable for characterizing protein–ligand complexes across a broad range of binding affinities (μM-nM) [11].

**Figure 4:**
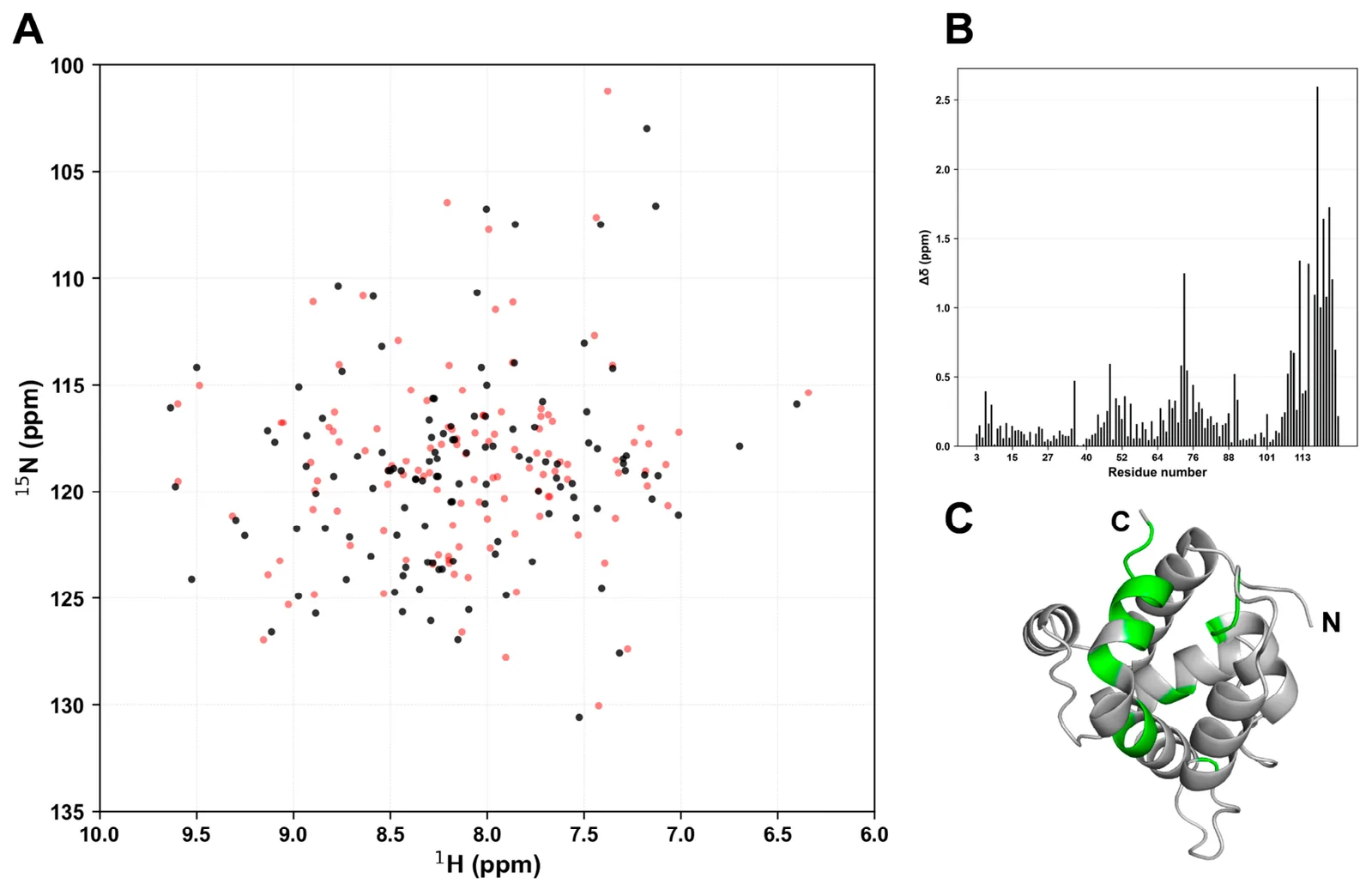
Ligand binding induces conformational changes in Drosophila OBP44a. (**A**) Superimposed ^1^H-^15^N HSQC spectra of free (black) and 8(Z)-eicosenoic acid-bound (red) OBP44a, showing significant chemical shift perturbations (CSPs) upon binding. (**B**) CSP values (Δδ = [(Δδ^1^H)^2^ + (Δδ^15^N/5)^2^]^1/2^) plotted against residue number, identifying the binding interface primarily in the C-terminal region. Data from BMRB entries 52374 and 52377 [31]. (**C**) Mapping of residues with Δδ > 0.5 ppm (green) onto the alphafold-predicted structure of OBP44a (via Swiss-Prot: AF-Q7K084-F1), highlighting the ligand binding site.

Chemical shift perturbation (CSP) is widely used in biological research. A series of ^1^H-^15^N HSQC spectra are acquired throughout the titration using a ^15^N-labeled protein concentration as low as 20 μM. For each titration point, following backbone assignment, peak positions are precisely quantified, and the combined chemical shift perturbation (Δδ) is calculated (Figure 4) [30,31]. By mapping the residues with chemical shift changes onto the three-dimensional structure of the protein, identifying which residues are affected by protein–ligand interaction is straightforward. Moreover, when the exchange between free and bound states is fast on the NMR chemical shift timescale, the concentration-dependent changes in chemical shift can be analyzed in two steps. Initially, individual fitting of the titration data for each affected residue provides a set of residue-specific apparent *K*_d_ values; mutual consistency among these values within experimental error suggests a single-site binding model, whereas significant deviations may indicate multiple binding sites or allosteric effects. Subsequently, global fitting of all residues simultaneously to a single binding isotherm yields a highly accurate global dissociation constant, while the CSP magnitude of each individual residue reflects its local environmental response to ligand binding [11,32].

In addition to NMR chemical shift perturbation analysis, we should also focus on signal intensity changes in ^1^H-^15^N HSQC spectra upon titration. In HSQC titration experiments, the intensity of a cross peak is not merely a reflection of concentration but is modulated by the apparent relaxation properties of the nucleus, which are sensitive to molecular dynamics and binding kinetics. The most fundamental information derived from intensity changes concerns the exchange regime on the NMR chemical shift timescale. The behavior of peak intensity during titration directly reports on the relationship between the kinetic rate constants and the chemical shift difference between the free and bound states [10,33].

Unlike ligand-observed methods, although CSP and intensity analyses are informative, the protein-observed ^1^H-^15^N HSQC is limited by protein size. For proteins larger than 30 kDa, spectral overlap and relaxation-induced line broadening severely attenuate signals [34,35]. Transverse relaxation-optimized spectroscopy (TROSY) reduces relaxation induced line broadening to improve spectral quality for these larger proteins [36]. However, TROSY is typically employed in conjunction with deuterated sample preparation, which increases the cost and sample preparation demands.

### 3.2. Site-Specific Labeling Strategies

For conventional ^1^H-^15^N HSQC, the upper limit is approximately 30 kDa for uniformly labeled proteins [34]. This barrier excludes a vast array of biologically important systems from routine analysis, including multi-domain proteins exceeding 30 kDa, and membrane proteins requiring detergent environments. Site-specific labeling strategies effectively overcome these molecular weight limitations, thereby enabling NMR characterization of large multi-domain proteins and membrane proteins [14,16,35]. Furthermore, this approach is applicable for characterizing protein–ligand interactions across a wide range of binding affinities, from weak (mM) to tight (nM) complexes [37,38].

In site-specific labeling strategies, ^19^F NMR offers distinct advantages that complement and extend beyond conventional approaches. The 100% natural abundance and high gyromagnetic ratio of ^19^F provide sensitivity approaching that of protons, enabling rapid data acquisition with simple pulse sequences [39]. While conventional heteronuclear NMR experiments on large proteins often require millimolar concentrations, ^19^F NMR can generate high-quality 1D spectra with protein concentrations as low as mid-micromolar [40]. The zero-background property means that spectra of fluorinated proteins exhibit no interfering signals from the surrounding environment. ^19^F can be applied to probe protein folding within the 2.5 MDa ribosome complex, facilitating the detection of transient folding intermediates [41].

In protein–ligand interaction studies, ^19^F NMR can be implemented through two complementary strategies: protein-observed approaches, where fluorinated reporters are incorporated into the target protein, and ligand-observed approaches, where fluorinated fragments or compounds are screened against unlabeled target proteins [40].

In the protein-observed ^19^F NMR approach, the successful application of ^19^F NMR to large protein systems critically depends on the method used to introduce fluorine reporters at defined positions. Two principal strategies have been developed for site-specific fluorine incorporation, each with distinct advantages and experimental considerations: (1) cysteine-based conjugation with fluorine-containing small molecule probes, and (2) biosynthetic incorporation of fluorinated amino acids during protein expression. Both approaches enable the introduction of fluorine probes at predetermined locations, yielding simplified 1D ^19^F spectra with resonances that report directly from sites of biological interest [42]. The utility of protein-observed ^19^F NMR has been demonstrated in membrane protein research; for example, Liu et al. utilized this approach on the β2-adrenergic receptor to reveal conformational ensembles underlying biased signaling [43]. Yuan et al. used ^19^F NMR to monitor the phosphoantigen (HMBPP)-induced conformational changes in BTN3A1 upon its association with BTN2A1, demonstrating the high sensitivity of this technique in detecting dynamic protein and small molecule interactions [44].

In the ligand-observed ^19^F NMR approach, fluorinated compounds are screened against unlabeled protein targets. The fundamental premise is simple: upon protein binding, the ^19^F NMR signal of a small molecule ligand undergoes changes in its chemical shift, linewidth, or intensity. This strategy has been successfully applied to GPCR ligand discovery; for instance, Zhang and colleagues developed a ligand-observed ^19^F NMR competition assay for the adenosine A_2_A receptor using a fluorinated probe molecule, FPPA, enabling the identification of diverse ligands without protein labeling [45].

Another widely used site-specific labeling strategy is ^13^C-methyl labeling, which is a protein-observed method. This approach introduces ^13^C-labeled methyl groups into selected amino acids including methionine, alanine, isoleucine, leucine, threonine, and valine, which enables NMR studies of large proteins such as GPCRs [46,47]. This technique is commonly achieved by expressing the target protein using isotopically labeled amino acid precursors in D_2_O-based medium. By recording ^1^H-^13^C HSQC spectra, researchers can observe chemical shift perturbations or signal intensity changes induced by ligand binding. This strategy has been effectively demonstrated on the atypical chemokine receptor 3 (ACKR3), where site-specific ^13^C-methyl methionine labeling combined with mutagenesis enabled the probing of individual residues to monitor ligand-induced conformational changes and signaling biases [17].

## 4. From Constraints to Coordinates: Determining Atomic-Resolution Structures

### 4.1. Conventional NMR Structures of Protein–Ligand Complexes

**Figure 5:**
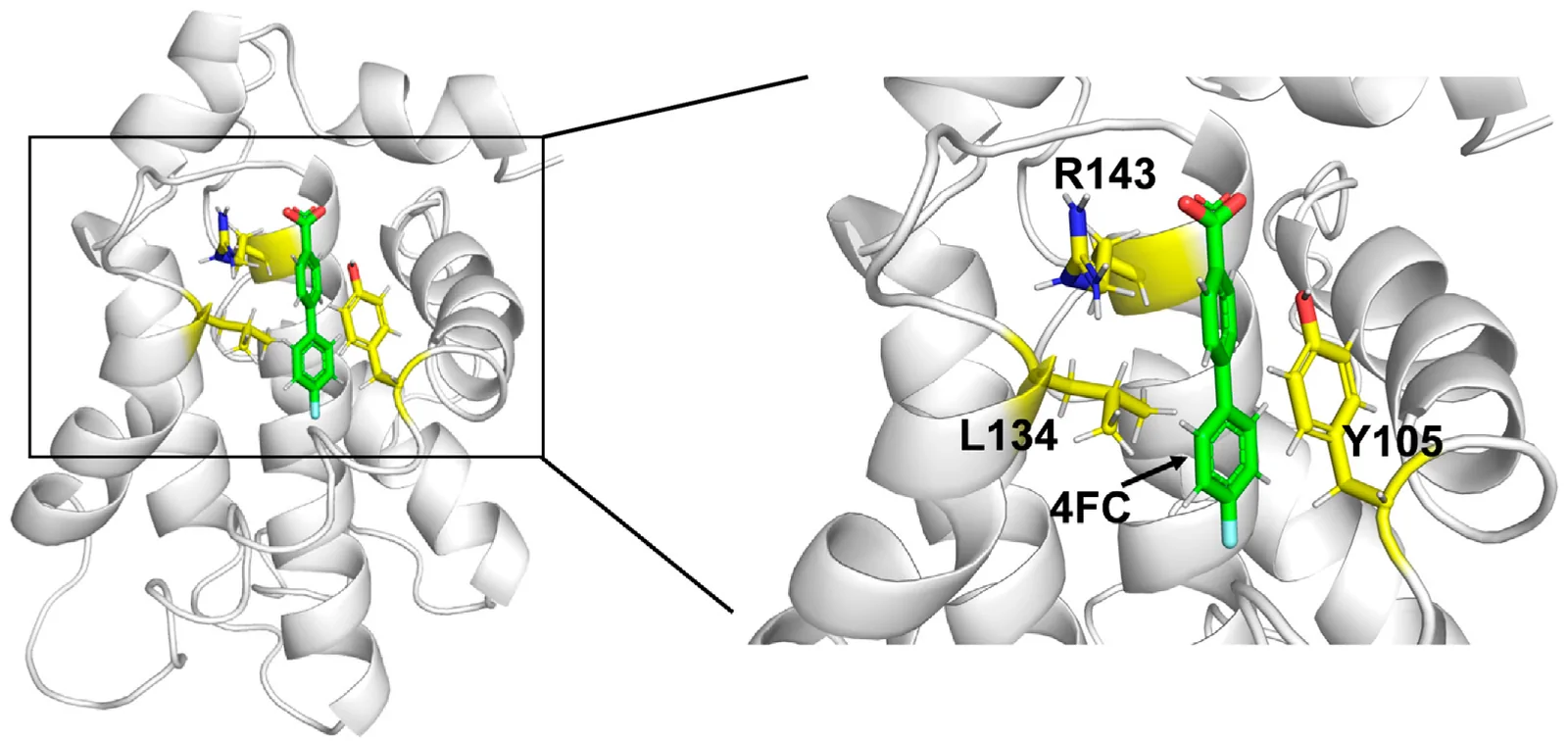
Structural characterization of the Bcl-xL ternary complex (PDB: 1YSG). Left panel: Overall structure of the complex. Right panel: Magnified view of the local region (indicated by the box in the left panel), showing the residues R143, L134, Y105 (yellow) close to small-molecule ligand 4′-Fluorobiphenyl-4-carboxylic acid (green). The structural model is restrained by 48 intermolecular protein–ligand NOEs. This structure shows the important role of NMR spectroscopy in elucidating the atomic details of protein–ligand interactions.

NMR is one of the three biophysical techniques for determining biomolecular structures at atomic resolution, and it is unique in its ability to determine three-dimensional structures under near-physiological solution conditions. The foundation of NMR structure determination is based on the nuclear Overhauser effect (NOE), which arises from dipole–dipole cross-relaxation between spatially close nuclei. The intensity of an NOE cross-peak is related to the inverse sixth power of the internuclear distance, making it sensitive for measuring ^1^H-^1^H distances up to approximately 5–6 Å [48,49]. For complexes with molecular weights below approximately 30 kDa, conventional multi-dimensional NMR experiments can be employed to generate distance restraints for structure calculation (Figure 5).

Intermolecular NOEs between protein and ligand protons are critical for structure determination of protein–ligand complexes, because these intermolecular NOEs provide direct restraints for defining the binding interface and orienting the ligand within the protein binding pocket. Intermolecular NOEs are typically obtained using isotope-filtered NOESY experiments that selectively detect correlations between protons attached to different isotopes, thereby suppressing intramolecular signals and revealing only the intermolecular contacts [50].

The disadvantage of structure calculation by NMR is that it is very time-consuming since all atoms in the complex need to be assigned to their respective chemical shifts. To address this, Orts et al. developed *N*MR^2^ (NMR Molecular Replacement), a method applicable to weak (dissociation constant, *K*_d_ ~ mM) and strong (dissociation constant, *K*_d_ ~ nM) binders across fast or slow exchange regimes, except in the cases of severe exchange broadening [51]. This innovative approach circumvents the need for protein resonance assignment by using a known protein structure combined with a set of intermolecular NOE restraints, thereby enabling the rapid and accurate elucidation of complex structures. *N*MR^2^ drastically reduces the determination time from several months to merely a few days. The method requires a minimum of approximately 12–15 intermolecular NOEs and relies on the availability of an input structure [51]. Accurate chemical shift assignment of the ligand in the bound state is needed before structure calculation. By integrating selective methyl protonation with the *N*MR^2^ methodology, Scanlon et al. rapidly determined the binding mode of the weak-affinity ligand phenylthiazole 1 (*K*_d_ ≈ 0.9 mM) to the 21 kDa enzyme EcDsbA without full protein resonance assignment [52].

### 4.2. NMR Structure Determination of Protein–Ligand Complexes by Specialized Strategies

Otting et al. successfully resolved the high-resolution structure of the 30 kDa thymidine–*E. coli* ϵ186/θ complex (K_d_ ≈ 7.6 mM) via transferred pseudocontact shifts (PCSs) [53]. This method relies on the presence of a lanthanide ion in the protein target and on fast exchange between bound and free ligand. By converting PCS data into long-range spatial restraints relative to a lanthanide probe, this strategy circumvents the need for extensive resonance assignment or ligand isotopic labeling. Although the method demands a pre-existing high-resolution protein structure as a template, it provides a rapid solution for characterizing weak affinity complexes with dissociation constants in the low millimolar range.

Chemical shift perturbation (CSP) data can serve as constraints for structure calculations to determine the three-dimensional structure of protein–ligand complexes. The HADDOCK (High Ambiguity Driven DOCKing) approach is based precisely on this principle: residues exhibiting significant chemical shift perturbations are defined as “active residues,” and their surface-adjacent residues are defined as “passive residues.” These are then employed as ambiguous interaction restraints to drive the docking of the ligand into the protein’s binding pocket [54].

Conventional NOE methods and specialized strategies are employed complementarily to complete the structure determination of protein–ligand complexes. Depending on the protein system under investigation, this methodological integration has significantly broadened the applicability of NMR in protein–ligand interaction studies.

## 5. Ligand-Induced Protein Dynamics by NMR

Beyond detecting binding, mapping interfaces and complex structure determination, one of the distinctive strengths of NMR is its ability to characterize protein conformational dynamics in solution under near-physiological conditions. While X-ray crystallography and cryo-EM provide largely static snapshots, NMR can directly probe motions across a wide range of timescales, from picosecond backbone fluctuations to millisecond conformational rearrangements. This capability makes NMR uniquely suited to study phenomena such as induced fit, conformational selection, allosteric modulation, and ligand-induced loop immobilization.

The most widely used NMR techniques for this purpose are spin relaxation experiments. Heteronuclear ^15^N relaxation measurements (R_1_, R_2_, and steady-state heteronuclear NOE) report on fast internal motions on the ps-ns timescale, allowing for researchers to identify flexible loops, inter-domain hinges, and disordered termini. Changes in these parameters upon ligand binding can reveal the rigidification of dynamic regions. For example, Gryk et al. used ^15^N relaxation measurements to compare the backbone dynamics of free and Ins(1,4,5)P_3_-bound β-spectrin PH domain [55]. Their data showed that the ligand-binding loops (β1–β2 and β5–β6) exhibit significant internal mobility on the ps-ns timescale in the free state, whereas ligand binding restricts these motions, as reflected by increased NOE values.

For slower motions (µs-ms) that are often linked to biological function, relaxation-dispersion experiments such as CPMG or T_1ρ_ relaxation dispersion are the methods of choice. These experiments can detect and quantify weakly populated excited conformational states, providing kinetic and thermodynamic information on conformational exchange processes. A seminal example is the work of Ishima et al., which systematically compared the backbone dynamics of free and DMP323-bound HIV-1 protease using combined ^1^H and ^15^N transverse relaxation experiments [56]. Their data revealed that the flap tips (residues 48–55) undergo a distinct conformational exchange on the ~80 µs timescale in the free enzyme, whereas this slow motion is largely suppressed upon inhibitor binding, directly demonstrating ligand-induced flap immobilization. In another classic study, Boehr et al. applied CPMG relaxation dispersion to each intermediate of the *E. coli* DHFR catalytic cycle and demonstrated that each intermediate samples low-lying excited states whose conformations resemble adjacent intermediates [57]. The hydride transfer and turnover rates are governed by transitions between these states, showing that ligand binding modulates the energy landscape to channel the enzyme along a preferred kinetic path.

Additional complementary tools include chemical exchange saturation transfer (CEST) for slow-exchange processes and ZZ-exchange spectroscopy for characterizing interconversion between distinct conformers. When the protein of interest is large or otherwise challenging for conventional ^1^H/^15^N methods, ^19^F NMR, which requires only a fluorine label and suffers no background signal, provides a sensitive alternative for monitoring conformational equilibria and ligand-induced changes in large proteins or complexes. For example, Overbeck et al. developed a consistent suite of ^19^F-based CPMG, on-resonance R_1ρ_ and off-resonance R1ρ relaxation dispersion experiments and successfully applied them to quantify µs-ms dynamics in the 360 kDa half-proteasome, demonstrating the feasibility of ^19^F NMR for studying large macromolecular assemblies [58].

The primary limitations of these dynamics-oriented NMR experiments typically involve the need for isotope labelling (^15^N and often ^13^C), the sensitivity constraints imposed by the protein’s molecular weight, and the complexity of data analysis for quantitative modelling. For proteins above approximately 30 kDa, deuteration and TROSY are generally necessary to achieve adequate resolution and sensitivity in conventional ^1^H/^15^N experiments (with the notable exception of ^19^F-based approaches, which bypass these requirements). Furthermore, relaxation dispersion experiments rely on exchange occurring in a suitable timescale window, and extremely slow or extremely fast motions may escape detection. Nevertheless, when applicable, these methods deliver mechanistic information on protein flexibility and conformational changes that cannot be obtained from static structural methods. Integrating these dynamic insights with binding data and high-resolution structures produces a richer, more complete picture of protein–ligand recognition, and this synergy underlies the growing role of NMR in drug discovery.

## 6. Discussion

**Figure 6:**
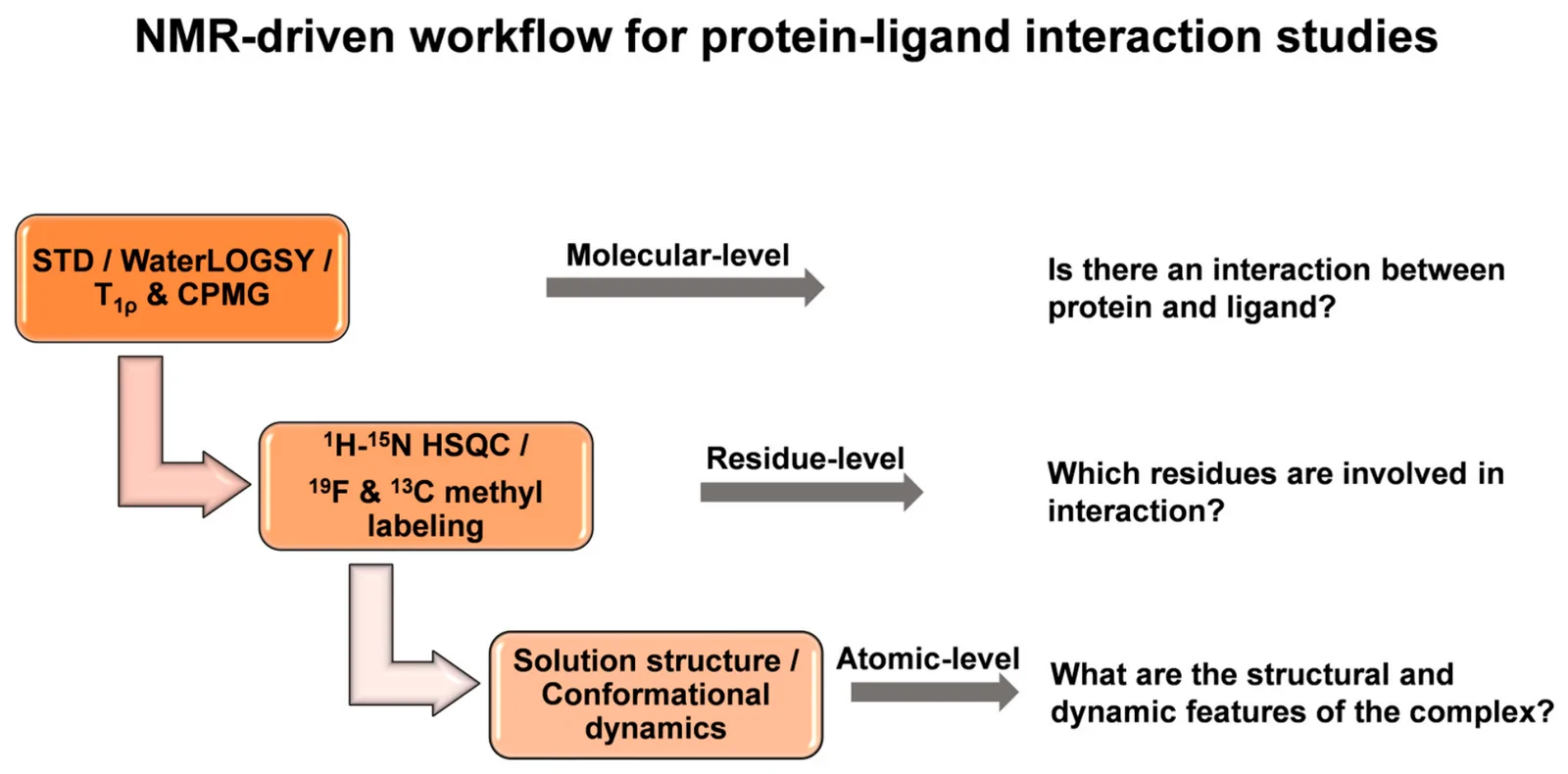
NMR-driven workflow for protein–ligand interaction studies. NMR offers a versatile toolkit for characterizing molecular recognition events. Based on the hierarchical information they provide, NMR experiments can be categorized into three distinct classes: (i) qualitative binding detection at the molecular level, (ii) mapping of binding interfaces at the residue level, and (iii) atomic-resolution structure determination and conformational dynamics characterization of protein–ligand complexes.

NMR provides a multi-scale platform for elucidating protein–ligand interactions, naturally organized into a progressive three-level workflow: rapid molecular-level binding detection, residue-level interface mapping, and atomic-level structure and dynamics characterization (Figure 6). This hierarchy enables an efficient transition from initial hit identification to mechanistic understanding of molecular recognition.

At the molecular level, ligand-observed experiments (STD, WaterLOGSY, relaxation-based methods) offer a rapid and sensitive filter for discriminating binders from non-binders with minimal protein consumption, establishing the essential starting point for any interaction study. Once binding is confirmed, protein-observed strategies including ^1^H-^15^N HSQC and site-specific labeling approaches can map the binding interface at residue resolution. Under favorable exchange conditions, they may also provide quantitative affinity estimates. These two stages together define whether and where an interaction occurs, providing the foundation for subsequent high-resolution investigation.

The third level exploits NMR’s unique ability to determine solution structures of protein–ligand complexes and to characterize conformational dynamics across multiple timescales. By integrating NOE-derived distance restraints, CSP-driven docking, and specialized approaches such as transferred PCS, atomic-resolution models of the binding interface can be obtained under near-physiological conditions, complementing static structures from X-ray crystallography and cryo-EM. Simultaneously, spin relaxation and relaxation dispersion experiments resolve motions ranging from picosecond backbone fluctuations to millisecond conformational exchanges, revealing functionally critical phenomena such as induced fit and allosteric modulation.

Each experimental level has intrinsic strengths and limitations that dictate its applicability to a given system (Table 1). Ligand-observed methods are effective in high-throughput screening but are largely restricted to weak-to-moderate affinity ranges; protein-observed and structural approaches are applicable across a wide affinity range, from weak to tight binding, but generally face molecular weight and sample preparation constraints. A notable exception is ^19^F NMR, which effectively circumvents the molecular weight limitation. A rigorous workflow therefore demands an integrated, hierarchical strategy in which the findings at each level guide the next steps, with complementary biophysical techniques (e.g., ITC, HDX-MS) called upon where necessary to validate or extend the NMR findings [59,60].

In summary, the hierarchical NMR framework described here converts a set of disparate experiments into a coherent, efficient pipeline for mapping protein–ligand interactions. By systematically advancing through detection, localization, and structural/dynamic characterization, this workflow not only provides a comprehensive view of molecular recognition but also offers a practical framework for both fundamental biological studies and drug discovery programs.

Looking forward, ongoing advances in computational and instrumental methods are expected to further strengthen this hierarchical framework. For example, deep learning algorithms have shown promise in predicting protein chemical shifts, which can reduce the manual effort required for spectral analysis [61]. The advent of 1.2 GHz NMR spectrometers offers substantially improved resolution and sensitivity, enabling atomic-resolution studies of more challenging systems, including large protein–ligand complexes. Continued progress in these areas is likely to broaden the scope of tractable targets and accelerate the transition from binding detection to atomic-level characterization.

## Figures and Tables

**Table 1:** NMR techniques for screening and characterizing protein–ligand interactions.

Level	Technique	Principle	Advantages	Limitations
Molecular level	STD (Saturation Transfer Difference)	Ligand-observed. Saturation is transferred from the protein to the bound ligand via spin diffusion. The difference spectrum reveals binding epitopes.	1. Effective for weak to moderate affinity (*K*_d_ in µM-mM range). 2. Works with low protein concentrations (µM range). 3. No isotopic labeling required.	1. Not suitable for tight binding (slow exchange regime). 2. Prone to false positives from non-specific binding or ligand aggregation. 3. Spin diffusion may overestimate binding epitope.
	WaterLOGSY (Water–Ligand Observed via Gradient Spectroscopy)	Ligand-observed. Magnetization is transferred from bulk water to the ligand via the protein. Bound ligands typically exhibit signal sign opposite to non-binders.	1. Highly sensitive for detecting weak binders (*K*_d_ in µM-mM range). 2. Works with low protein concentrations (µM range). 3. No isotopic labeling required.	1. Requires ligand protons for detection; signals may be weak or absent for certain ligand classes. 2. Susceptible to artifacts from non-specific binding or ligand aggregation.
	CPMG/T_1ρ_	Ligand-observed. Bound ligand tumbles more slowly, enhancing transverse relaxation and attenuating signal in CPMG/T_1ρ_ spectra; comparison of spectra with and without protein identifies binders. In the slow-exchange limit (tight binding), signal attenuation can become complete, which itself confirms binding.	1. Applicable to a wide affinity range (weak to tight) with optimized ratios. 2. Works with low protein concentrations (µM range). 3. No isotopic labeling required.	1. Optimization of ligand/protein ratios is critical: for weak binders, excess ligand is needed for detectable attenuation; for tight binders, 1:1 ratio may be used, where signal loss (rather than attenuation) reports binding. 2. False positives may arise from non-specific binding or ligand aggregation.
Residue level	^19^F NMR	Ligand- or Protein-observed. Monitoring chemical shift perturbations or line-broadening of fluorine nuclei. High sensitivity due to 100% natural abundance and lack of background.	1. Broad *K*_d_ range (nM-mM); applicable to both weak and tight binding. 2. Applicable to large proteins.	1. Requires incorporation of fluorine (synthesis of fluorinated ligands or biosynthetic labeling). 2. Limited to fluorine-containing systems.
	^1^H-^15^N HSQC	Protein-observed. Monitoring chemical shift perturbations (CSPs) of backbone amide resonances upon ligand titration.	1. Directly reveals the binding interface; suitable for both weak and tight binding. 2. With TROSY/deuteration, applicable well above 30 kDa.	1. Requires ^15^N labeling. 2. Protein size typically <30 kDa without TROSY. 3. Needs relatively high protein concentration and stability (typically tens to hundreds of µM).
Atomic level	Structure and Dynamics (NOE, Relaxation)	Protein/ligand-observed. NOE-based distance restraints, structure calculation; relaxation parameters (R_1_, R_2_, steady-state NOE) for ps-ns dynamics; CPMG/R_1ρ_ dispersion and ZZ-exchange for µs-ms to slower exchange processes	1. Provides atomic-resolution binding mode and conformational ensembles. 2. Characterizes dynamics from ps-ns to ms timescales. 3. Near-physiological solution conditions.	1. Demands high protein stability, solubility, and concentration (~1 mM); 2. Size limited to <30 kDa 3. Time-consuming for data acquisition and analysis;

## Data Availability

No new data were created or analyzed in this study. Data sharing is not applicable to this article.

## Abbreviations

The following abbreviations are used in this manuscript:

NMR	Nuclear Magnetic Resonance spectroscopy. A technique that exploits the magnetic properties of certain atomic nuclei to determine the structure, dynamics, and interactions of molecules in solution.
STD	Saturation Transfer Difference spectroscopy. A ligand-observed NMR experiment in which saturation is transferred from the protein to the bound ligand; comparison of spectra with and without saturation reveals which ligand protons are in close contact with the protein.
WaterLOGSY	Water–Ligand Observed via Gradient Spectroscopy. A ligand-observed NMR experiment that detects binding through magnetization transfer from bulk water to the ligand, mediated by the protein. Useful for primary screening and competition experiments.
T_1ρ_	Spin-lattice relaxation in the rotating frame. A relaxation-based NMR experiment sensitive to exchange processes on the microsecond-to-millisecond timescale. Used to study dynamics and binding events.
CPMG	Carr–Purcell–Meiboom–Gill pulse sequence. A relaxation-based NMR experiment that measures transverse relaxation (T2) in both ligand- and protein-observed formats; used to study dynamics and binding events.
HSQC (^1^H-^15^N HSQC)	Heteronuclear Single Quantum Coherence spectroscopy. A two-dimensional protein-observed NMR experiment that correlates proton and nitrogen chemical shifts, producing a characteristic “fingerprint” of the protein backbone. It is widely used to detect ligand binding and map interaction interfaces.
CSP	Chemical Shift Perturbation. The change in NMR resonance frequencies of protein nuclei (typically amide groups) upon ligand binding. Used to map interaction sites on the protein and to measure binding affinity through titration experiments.
TROSY	Transverse Relaxation-Optimized Spectroscopy. An NMR technique that selects the slowly relaxing component of coupled spin systems, dramatically improving spectral resolution for high molecular weight proteins.
ITC	Isothermal Titration Calorimetry. A biophysical method that directly measures the heat released or absorbed upon binding, providing the dissociation constant (*K*_d_), stoichiometry, enthalpy, and entropy of a protein–ligand interaction.
HDX-MS	Hydrogen–Deuterium Exchange Mass Spectrometry. A technique that monitors the exchange of backbone amide protons with deuterium in solution; ligand binding protects certain regions from exchange, revealing binding sites and conformational changes.
